# Branched Chain Amino Acids: Beyond Nutrition Metabolism

**DOI:** 10.3390/ijms19040954

**Published:** 2018-03-23

**Authors:** Cunxi Nie, Ting He, Wenju Zhang, Guolong Zhang, Xi Ma

**Affiliations:** 1 State Key Laboratory of Animal Nutrition, College of Animal Science and Technology, China Agricultural University, No. 2. Yuanmingyuan West Road, Beijing 100193, China; niecunxi@shzu.edu.cn (C.N.); ht920819@cau.edu.cn (T.H.); 2 College of Animal Science and Technology, Shihezi University, No. 221. Beisi Road, Shihezi, Xinjiang 832003, China; zwj@shu.edu.cn (W.Z.); 3 Department of Animal Science, Oklahoma State University, Stillwater, OK 74078, USA; 4 Department of Internal Medicine, University of Texas Southwestern Medical Center, Dallas, TX 75390, USA

**Keywords:** branch chain amino acids (BCAAs), amino acid metabolism, PI3K-AKT-mTOR, biomarkers, insulin resistance, metabolic diseases

## Abstract

Branched chain amino acids (BCAAs), including leucine (Leu), isoleucine (Ile), and valine (Val), play critical roles in the regulation of energy homeostasis, nutrition metabolism, gut health, immunity and disease in humans and animals. As the most abundant of essential amino acids (EAAs), BCAAs are not only the substrates for synthesis of nitrogenous compounds, they also serve as signaling molecules regulating metabolism of glucose, lipid, and protein synthesis, intestinal health, and immunity via special signaling network, especially phosphoinositide 3-kinase/protein kinase B/mammalian target of rapamycin (PI3K/AKT/mTOR) signal pathway. Current evidence supports BCAAs and their derivatives as the potential biomarkers of diseases such as insulin resistance (IR), type 2 diabetes mellitus (T2DM), cancer, and cardiovascular diseases (CVDs). These diseases are closely associated with catabolism and balance of BCAAs. Hence, optimizing dietary BCAA levels should have a positive effect on the parameters associated with health and diseases. This review focuses on recent findings of BCAAs in metabolic pathways and regulation, and underlying the relationship of BCAAs to related disease processes.

## 1. Introduction

Amino acids (AAs) are best known as the nutrient substrates for protein synthesis; in addition, they can also participate as bioactive molecules in nutrition metabolism. Among them, the branched chain amino acids (BCAAs) refer to leucine (Leu), isoleucine (Ile), and valine (Val), and are important nutrition signals that have important mediation effects on protein synthesis, glucose homeostasis, anti-obesity, and nutrient-sensitive signaling pathways, e.g., phosphoinositide 3-kinase-protein kinase B (PI3K-AKT), mammalian target of rapamycin (mTOR) [1,2]. The results from a previous study have suggested that increased BCAAs catabolic flux may contribute to increased gluconeogenesis and glucose intolerance via glutamate transamination to alanine [3]. In addition, the levels of circulating BCAAs tend to be elevated in obesity individuals, and this increased circulating level of BCAAs has a close relationship with harmful metabolic health and future insulin resistance (IR) or type 2 diabetes mellitus (T2DM) [2,4]. Ruiz-Canela et al. [5] reported that high circulating BCAAs at the baseline were directly associated with risk of cardiovascular diseases (CVDs), and these deleterious relationships may be counteracted by means of Mediterranean-style diet interventions to alter pathophysiological processes, and, furthermore, to exert its cardioprotective effects. Therefore, BCAAs as biomarkers have been found to predict obesity, IR, T2DM, and CVDs outcomes [5,6]. Targeted therapies have increasingly focused on treating advanced malignancies via inhibition of the dysregulated signaling network, such as the PI3K-AKT-mTOR signal pathway [7,8]. Previous reports showed AAs can activate Akt signaling by class I PI3K, and this signaling pathway also leads to activation of mTORC2 and offering new insights into the role of this signaling pathway in growth, proliferation, and survival in cells [9]. More importantly, limited studies have indicated that BCAAs play essential roles of physiological regulation in many processes besides simple nutrition, especially the disease progression. This review provides recent findings on BCAAs metabolism, physiological functions, and outstanding signaling pathways, underlying mechanism to trigger disease and targeted therapy as biomarkers of IR, T2DM, cancer and CVDs.

## 2. Catabolism and Balance of BCAAs

**Figure 1:**
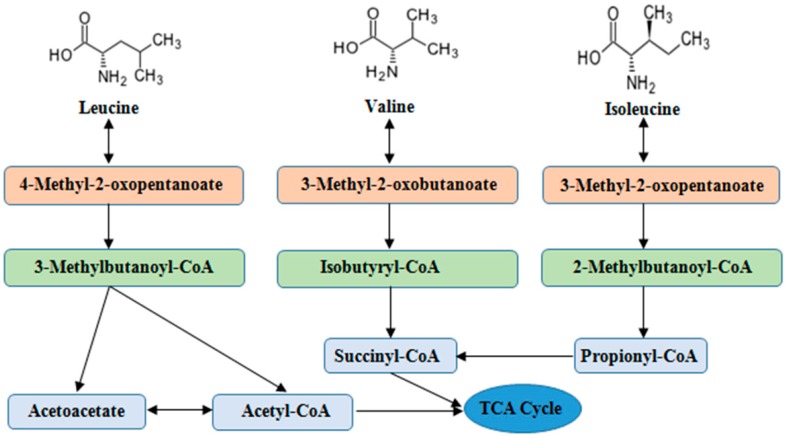
The main steps of BCAAs catabolism. BCAAs catabolic reactions are catalyzed via a series of enzymes (e.g., transamination by BCAT) and decarboxylation by BCKD), and the end products enter to TCA cycle. Abbreviations: BCAAs, branched chain amino acids; BCAT, branched-chain amino acid aminotransferases; BCKD, branched-chain α-keto acid dehydrogenase; TCA, tricarboxylic acid.

Unlike most other EAAs, BCAAs catabolism is initially catalyzed in extrahepatic tissues by the branched-chain amino acid aminotransferases (BCAT) and branched-chain α-keto acid dehydrogenase enzyme complex (BCKDC). After main reaction steps of transamination by BCAT and decarboxylation by BCKDC, the BCAAs metabolites are further converted to final products (acetyl-CoA and Succinyl-CoA) via a series of enzymatic reactions and participate in metabolism of tricarboxylic acid (TCA) cycle [10,11,12] (Figure 1).

Normally, the supply and consumption of AAs are equal including quantity and proportion. The excesses or shortages of AAs are closely related to protein metabolism, which can result in increased urea production or restriction of cells growth. Short-term malnutrition does not cause depletion of plasma AAs due to reduced protein synthesis and the onset of autophagy in body. Boutry et al. [13] reported that Leu pulses increased muscle protein synthesis during continuous feeding in neonatal pigs. Supplementing BCAAs in low protein diet (16.7%) regulated the arterial concentrations and intramuscular catabolism of BCAAs in young pigs [14]. Cellular AAs balance is regulated by the AAs signaling, enzymes, transporters, and tRNA molecules [15]. 

**Figure 2:**
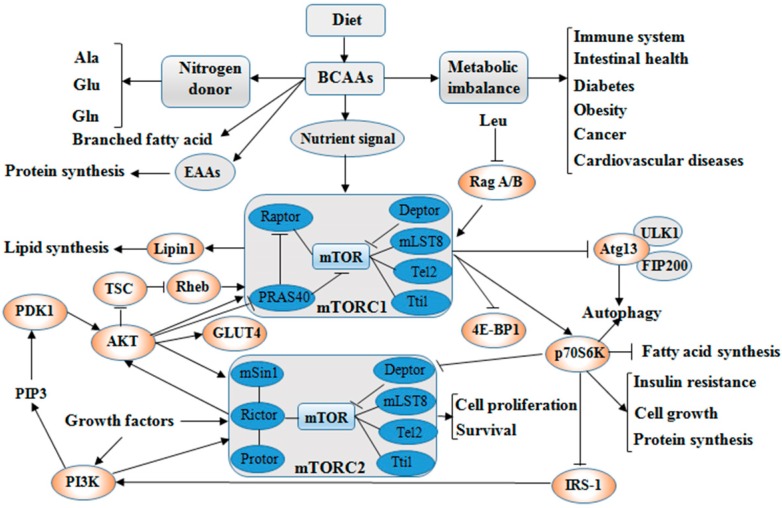
BCAAs balance and its multiple roles via PI3K-AKT-mTOR signaling pathway. BCAAs play important role as nitrogen donor for AAs, such as Ala, Glu, and Gln, and also as nutrient signal play critical roles in multiple metabolic functions through special signaling pathway, especially via PI3K-AKT-mTOR pathway. The metabolic imbalance of BCAAs can cause many health issues, such as diabetes and cancer. Abbreviations: AAs, amino acids; AKT, protein kinase B; Ala, alanine; Atg13, autophagy 13; BCAAs, branched chain amino acids; Deptor, domain containing mTOR interacting protein; 4E-BP1, 4E-binding protein 1; EAAs, essential amino acids; FIP200, focal adhesion kinase-interacting protein 200 kDa; Gln, glutamine; GLUT4, glucose transporter; Glu, glutamate; IRS-1, insulin receptor substrate 1; Leu, leucine; Lipin1, phosphatidate phosphatase Lipin1; mLST8, mammalian lethal with Sec13 protein 8 (also known as GβL), mSin1, target of rapamycin complex 2 subunit MAPKAP1; mTOR, mammalian target of rapamycin; mTORC1, mTOR complex 1; mTORC2, mTOR complex 2; p70S6K, p70S6 kinase; PDK1, 3-phosphoinositide dependent protein kinase-1; PI3K, phosphoinositide 3-kinase; PIP3, phosphatidylinositol-3,4,5-trisphosphate; PRAS40, proline-rich Akt substrate 40 kDa; Protor, proline-rich protein; Rag A/B, Ras-related GTP-binding protein A/B; Raptor, regulatory-associated protein of mTOR; Rheb, Ras homolog enriched in brain; Rictor, apamycin-insensitive companion of mTOR; Tel2, telomere length regulation protein; TSC, Tuberous sclerosis; Tti1, TELO2-interacting protein 1; ULK1, UNC51-like kinase 1. (The current understanding of the signaling pathway was based on annotations from the Kyoto Encyclopaedia of Genes and Genomes (KEGG)).

BCAAs exert responses to several cells signaling mainly through the activation of the mTOR axis [16]. The mTOR is the central regulator of mammalian cell growth [16], proliferation and migration [17], hypertrophy [18], IR [19] as well as pathogenesis of many other human diseases and survival, and is an evolutionary conserved serine/threonine protein kinase in the PI3K-related kinase (PIKK) family [20,21]. The mTOR pathway is the most well-known AAs sensor in maintenance of AAs balance, which is regulated by AA transporters, and/or biosynthesis and degradation of AAs and protein in cells. Apart from nitrogen donors for some AAs (alanine, glutamate, and glutamine), BCAAs act as a nutrient signal and play critical roles in multiple metabolic functions through special signaling especially via a PI3K-AKT-mTOR pathway (Figure 2). 

Dysregulation of BCAAs metabolism is associated with a range of diseases such as Maple Syrup Urine Disease (MSUD) caused by BCKDC dysfunction. The mutation of genes encoded BCKDC causes an inherited disorder in BCAAs catabolism and further results in the accumulation of BCAAs metabolites including ketoisocaproic acid (KIC), ketoisovaleric acid (KIV) and ketomethylvaleric acid (KMV) from Leu, Val and Ile, respectively [22]. In addition, the dietary protein sources with balanced AAs outstanding improved the performance, intestinal permeability and integrity of broiler chickens [23]. AAs deprivation induces protein scavenging despite persistent mTORC1 activity, and partial mTOR inhibition enhances cell growth by restoring AA balance reliant on eating extracellular protein [24]. Excess dietary Leu decreased growth performance, increased levels of plasma Leu and serum α-keto-isocaproate in a linear manner and BCAAs catabolism by means of posttranscriptional mechanisms [25]. However, Leu also regulates three BCAAs concentrations in muscle tissues, and the decline of Ile and Val concentrations may induce the activation of cardiac BCKDC [26]. The previous publication reported that the accumulation of mitotoxic metabolites (and not BCAAs per se) promotes β-cell mitochondrial dysfunction, stress signalling and apoptosis associated with T2DM in a BCAAs dysmetabolism model [2]. 

## 3. Roles of BCAAs in Nutrition Metabolism

### 3.1. Glucose and Lipid Metabolism

Apart from as nutritionally EAAs for protein synthesis, the BCAAs also as the signaling molecules participated in nutrition metabolism [27]. Adipose tissue plays a major role in glucose and lipid homeostasis through the storage of excess nutrients and lipolysis, and also has a role in maintaining balance of BCAAs. Excess nutrient intake or obesity causes both raise BCAAs catabolism and inhibition of fatty acid oxidation in the skeletal muscle and adipose tissue, respectively [28]. The previous study has demonstrated the potential capacity for adipose tissue to regulate circulating BCAAs in vivo via coordinate regulation of adipose-tissue BCAAs enzymes [29]. Another study reported that specifically reducing dietary levels of BCAAs have beneficial effects on the metabolic health in the young, growing mice, such as improve glucose tolerance, modestly slowing fat mass gain, and rapidly reverses diet-induced obesity [30]. The normalization of weight was regulated by increased energy expenditure, but not by caloric restriction or increased activity, and a transient induction of the energy balance regulating hormone fibroblast growth factor 21 (FGF21) play a critical role in this procession [30]. Glutamine as Leu metabolites plays a crucial role in various cellular processes, such as energy balance, apoptosis, and cell proliferation; it could activate the fatty acid β-oxidation pathway in HepG2 cells [31]. This result shows that glutamine deprivation can activate the fatty acid β-oxidation pathway to regulate lipid metabolism. 

BCAAs can regulate the metabolism of glucose and lipid via phosphatidylinositol 3-kinase (PI3K)-AKT (also referred to as protein kinase B, PKB) pathway [32]. The PI3K, as a nuclear factor, participate in many biological processes, and plays a critical role in cellular functions including immunity, growth, and survival in cellular signaling pathways [8,33,34]. The analysis of the signaling pathway indicated that Ile mediates the glucose uptake by PI3K, but was independent of mTOR [35]. Ile prevents the increase of plasma glucose concentration, stimulates glucose uptake in skeletal muscle, and also has an effect of prevention on the development of visceral obesity and hyperinsulinemia [36,37]. Compared with Leu and Val, Ile more significantly decreases the level of plasma glucose in an oral glucose tolerance test in normal rats [35]. BCAAs deficiency significantly changes lipid metabolism in white adipose tissue. Leu deprivation can suppress hepatic lipogenesis and increase fat mobilization in white adipose tissue (WAT), and Val or Ile deficiency has similar effects on reducing fat mass [38].

Dietary supplementation of Leu can inhibit the activation of AMP-activated protein kinase (AMPK), which is a signal sensor for maintaining energy homeostasis [39]. Low BCAAs levels suppress fatty acid synthesis and improve fatty acid β-oxidation by modulating the hepatic lipogenic gene expression in female broiler chickens, and this modulation is likely conducted through the AMPK-mTOR-FoxO1 pathway [40]. The optimal BCAAs ratio (Leu:Ile:Val = 1:0.75:0.75–1:0.25:0.25) added in a restricted protein diet (17% CP) could increase the uncoupling protein 3 (UCP3) mRNA level in the oxido-glycolytic skeletal muscle. The abundance of UCP3 is closely related to glucose metabolism in skeletal muscle, and UCP3 influence glucose uptake through glucose transporter 4 (GLUT4) translocation [41]. In addition, branched-chain α-keto acids (BCKA), a metabolite of BCAAs, inhibit mitochondrial respiration and energy metabolism in neuronal cells, but can protect mitochondria and energy production against oxidative injury [42,43]. β-hydroxy-β-methylbutyrate (HMB) might also regulate lipid metabolism [44], but lack of detailed results support it. Furthermore, addition of glutamine (BCAAs metabolite) to regimen of liraglutide in diabetic rats enhances insulin production and hence the glycemic control, which was associated by an upregulation in the expression of sodium-dependent neutral amino acid transporter-2 (transport glutamine for regulating insulin and glucagon secretions) in the pancreas [45]. Further results indicate that varying BCAAs ratios could regulate fatty acid synthesis, transport, oxidation, lipolysis, and adipokine secretion, which is related to the genes’ expression of adipose tissue function such as AMPKα, mTOR, silent information regulator transcript 1 (SIRT1) and peroxisome proliferator-activated receptor-g coactivator-1α (PGC-1α) [46]. These effects may be modulated via the AMPK-mTOR pathway, Sirt1-AMPK-PGC-1α axis and mitochondrial biogenesis. Krüppel-like factor 15 (KLF15), a transcription factor, plays a key function in regulating glycemic, lipid, and AAs metabolism of numerous cells, especially in BCAAs metabolism [47]. A recent study found that the high concentrations of BCAAs suppressed KLF15 expression while BCAAs starvation induced KLF15 expression [32]. 

### 3.2. Protein Synthesis

BCAAs stimulate protein synthesis in vitro preparations of skeletal muscle, in which Leu plays the most important roles, and this stimulatory effect is mediated by upregulating the initiation of mRNA translation including TSC2, Rheb, and raptor in the mTOR pathway [48]. However, insulin may be needed for the Leu-induced in vivo stimulation of protein synthesis in skeletal muscles [49]. Apart from skeletal muscle, Leu also enhances protein synthesis in other tissues such as adipose tissue [50]. Ile or Val alone has no effectiveness as a nutrient regulator of protein synthesis in skeletal muscles [48]. Orally administered Leu stimulates mTOR signaling and promotes phosphorylation of both 4E-BP1 and S6K1, but does not enhance global rates of protein synthesis in liver [51]. Low doses of Leu supplementation were found to enhance fat loss and effectively stimulates muscle protein synthesis in food-restricted rats [52]. Supplemented with a high (5.0 g total Leu) amount of Leu in a low-protein (6.25 g) mixed macronutrient beverage can stimulate myofibrillar muscle protein synthesis as effective as a high-protein dose (25 g) in the men [53]. Leu supplementation could stimulate muscle protein synthesis by activating the mTOR signaling pathway, especially Leu, who produced via lysosomal proteolysis could activate mTORC1 [54]. Leu also increased muscle protein synthesis by stimulating translation initiation [13]. Furthermore, a recent publication reported that supplementation with BCAAs in low protein diets can increase the net AAs fluxes across skeletal muscle in vivo. This elevated net AAs fluxes were related to the increase concentrations of BCAAs in arterial and intramuscular plasma and venous metabolites including BCKA and free fatty acids, and were also associated with the decrease content of 3-methylhistidinein in the biceps femoris muscle [14]. 

Numerous studies have demonstrated that Leu has critical biochemical actions involved in stimulating protein synthesis, inhibiting protein degradation, augmenting the activity of protein and availability of specific eukaryotic initiation factors [55,56]. BCAAs, particularly Leu, modulate partly the function of proteins in mRNA translation via activating the mTOR signaling pathway including the phosphorylation of S6K1, 4E-binding protein 1 (4E-BP1) and Eukaryotic initiation factor 4E (eIF4E) assembly [48,57]. An investigation showed that muscle myofibrillar protein synthesis was 22% higher in BCAAs ingestion alone (i.e., without other concurrent ingestion EAA, intact protein, or other macronutrients) then a placebo ingestion, and have a greater phosphorylation status of S6K1 and PRAS40 via activating cell mTOR signaling pathways following resistance exercise in humans [58]. The presence of 7.5 mM Leu could enhance the mRNA expression of the Na^+^-neutral AA exchanger 2 (ASCT2), and cause an increase in ASCT2 protein expression. Leu also activated phosphorylation of 4E-BP1 and eIF4E through the phosphorylation of PI3K-AKT-mTOR, and ERK signaling pathways in IPE-J2 cells, but isoleucine or valine could not [59]. A recent prospective study reported that low-protein diets of growing pigs supplemented with optimal BCAAs ratio (1:0.75:0.75–1:0.25:0.25) induce muscular protein metabolism, which is likely attributed to the activation of the AMPK-SIRT1-PGC-1α axis [41]. 

Small doses of Leu administration regulate skeletal muscle protein synthesis through multiple biomarkers of mRNA translation initiation, i.e., eIF 4E-BP1, phosphorylation of the 70-kDa ribosomal protein S6 kinase (p70S6K) [52]. Through its downstream effector, p70S6 kinase and direct target 4E-BP1 regulate protein translation. Leu depletion directly caused the Sestrin2-GATOR2 interaction, and Leu must be able to bind Sestrin2 before activating mTORC1 in cells [60]. Leucyl-tRNA synthetase (LeuRS), a direct sensor for Leu upstream of mTOR, is a cytoplasmic enzyme and is required for protein synthesis [1]. Intracellular Leu concentration is sensed by LeuRS, which is a key mediator for AA signaling to mTORC1 and induces mTORC1 activation via directly binding to Rag GTPase [61].

BCAAs metabolites such as BCKA, HMB and glutamine play an important role in protein synthesis. A recent study has shown that BCKA can significantly decrease protein expression of mTORC2 component (rictor) in cardiomyocytes [62]. Leu metabolite HMB has a significant increase of muscle protein synthesis and decrease muscle protein breakdown, and a large single oral dose (~3 g) of Ca-HMB (calcium salt of HMB) robustly (near maximally) stimulates skeletal muscle anabolism via mTORC1 [63]. The HMB stimulates protein synthesis through upregulation of mTOR signaling pathways, and HMB was much more effective than Leu in increasing protein synthesis through the mTOR system in rat L6 myotubes [64]. Moreover, intravenous infusion of HMB with increasing doses in neonatal piglets for one hour leads to mTOR activation and an increase in muscle protein synthesis [65]. HMB supplementation also increased skeletal muscle protein anabolism in neonates by stimulation of protein synthesis and satellite cell proliferation [66]. A novel study reported that HMB enhances the gain of skeletal muscle mass by increasing protein synthesis or/and attenuating protein degradation, and improves protein anabolism in muscles by increasing phosphorylation of protein anabolic molecules [67]. Aversa et al. reported that HMB administration in vivo Wistar rats model of cancer cachexia markedly increased the phosphorylated p70S6K and phosphorylated mTOR, and remarkable increased phosphorylated 4-E-BP1 was alleviated in rats received an i.p. inoculum of AH-130 cells by HMB treatment [68]. Similar results of protein degradation indirectly affected by BCAAs were reported [2]. BCAAs represent the major nitrogen source for glutamine, which has an important function in enhancing AAs synthesis, and acts a “nitrogen shuttle” among organs [69]. 

## 4. Physiological Functions of BCAAs on Intestinal Health and Immunity

### 4.1. Intestinal Health 

AAs have been focused on gastrointestinal diseases in human and animal as therapeutics, for example, inflammatory bowel disease (IBD), and diarrhea. As an important organ in the body, the intestinal tract has the highest level of immune activity and the destruction of intestinal homeostasis is closely related to the development of obesity, T2DM, IBD, atherosclerosis, and colon cancer [70,71,72]. Growing investigations with animals and humans indicate that AAs are key regulators in maintaining intestinal health besides being substrates for the protein synthesis and other nitrogenous compounds, as well as the roles in maintenance and growth of organisms [73,74]. For example, glutamine, arginine, and threonine could improve integrity of tight junctions, cell migration, anti-oxidative responses, and mucosal barrier functions in the intestine [74]. Likewise, BCAAs also act as a regulator to promote intestinal development, nutrient transporters, and immune-related function and then improve gut health [75,76,77]. However, most of the studies focus on the Leu functions but not Val or Ile in the intestine. Leu supplementation can maintain intestinal health by enhancing tight junction in fish [78] as well as improve the cell proliferation of intestinal epithelial, villus height and small intestinal growth of pigs, but the intestinal growth was inhibited when the level of Leu up to 2.57% [75]. The mRNA expressions of mTOR, and p70S6K in the jejunum and ileum were elevated with the increasing dietary Leu level from 1.37% to 2.17%, which indicates that the Leu administration promotes intestinal development through stimulating the activation of mTOR and its downstream pathway [75]. For nutrients such as fiber, AAs can modulate the intestinal microbiota, which also play an important role in host health [77]. Elevated systemic concentrations of certain AAs provided by gut bacteria, in particular BCAAs, have effects in modulating the development of IR and T2DM [79,80]. Dietary Ile improves microbial population in the intestine of juvenile Jian carp (Cyprinus carpio var. Jian). With graded Ile levels, *Lactobacillus* count exhibited a linear response, and the populations of *Bacillus*, *Aeromonas* and *E. coli* showed quadratic response [81]. Intestinal *Bacillus* was maximum for fish fed 13.9 g/kg Ile diet, and the counts of *Aeromonas* and *E. coli* were the lowest for fish fed the 11.9 g/kg Ile diet [81].

Besides regulating intestinal development and intestinal AAs transporter expression, BCAAs also have an intimate connection with other intestinal functions. BCAAs may enhance disease resistance and intestinal health through modulation of endogenous defensin in young animals and children. Ren et al. [76] investigated the mRNA expression of porcine epithelial β-defensins in response to BCAAs. The results indicated that BCAAs treatment increased the expression of β-defensins in jejunum and ileum of weaned piglets, and the similar stimulate effect of BCAAs on β-defensin expression was found in IPEC-J2 cells. In addition, the high enzyme expression BCAAs metabolism such as BCAT and branched-chain α-keto acid dehydrogenase (BCKD) in the intestine indicates the strong connection between BCAAs and intestinal function [12].

### 4.2. Immunity

In recent years, investigators have focused on the effects of BCAAs on the immune functions. Immune cells could incorporate BCAAs into proteins, express branched-chain alpha keto acid dehydrogenase and decarboxylase activities and are able to oxidize BCAAs [82]. Ile is greatest in lymphocytes, followed by eosinophils and neutrophils successively, and Leu can promote its own degradation by increasing the activity of lymphocyte branched-chain keto acid dehydrogenase [82]. BCAAs, as donors of nitrogen and of carbon skeletons for the synthesis of other amino acids like glutamine, are important in supporting immune cell function [83]. BCAAs’ deficiency of diet impairs the innate immune function due to the shortage of lymphocytes and white blood cells, and increases susceptibility to pathogens [84]. BCAAs supplementation could restore host defense mechanisms, namely phagocytic function of neutrophils and natural killer cell activity in cirrhotic patients [85]. Moreover, BCAAs can stimulate the SIgA secretion to enhance the mucosal surface defense, and it can also inhibit pathogen introgression into the lamina propria to improve the host immunity [84]. 

BCAAs regulate immune functions including increase fuel sources for immune cells, CD4+, CD4+/CD8+, intestinal immunoglobulins, innate and adaptive immune responses, pro-inflammatory cytokines, and dendritic cell function [12]. Intratracheal administration of Ile induced a significant increase of β-defensins 3 and 4 associated with decreased bacillary loads and tissue damage, which indicated induction of β-defensins by Ile as novel immunotherapy in infectious disease [86]. In addition, BCAA supplementation recovers the ability of peripheral blood mononuclear cells proliferate in response to mitogens after a long distance intense exercise associated with immunosuppression, as well as plasma glutamine concentration, which affects natural killer cells, lymphokine-activated killer cells, and lymphocytes [87]. The depletion of Val or Leu decreased p70S6K expression, and Val increased dose-dependently the allostimulatory capacity and IL-12 production of dendritic cells from both healthy volunteers and hepatitis C virus (HCV) cirrhotic patients [88]. Dietary Ile significantly increased the relative mRNA expression of transforming growth factor β2 (TGF-β2) and TOR in the head kidney with increasing Ile levels to improve the fish immune response [89], and have a downward trend in the relative mRNA expression of occludin, claudin-3, claudin-7, tumor necrosis factor-α (TNF-α), interleukin 10 (IL-10), Kelch-like-ECH-associated protein 1 (KEAP1), and extracellular signal-regulated kinase 1 (ERK1) in the fish intestine [81]. Investigation of enteral nutrition with parenteral glutamine supplementation indicated that glutamine can diminish the release of inflammatory cytokines, attenuate lymphatic organ apoptosis, and improve the immunological function in septic rats [90]. A recent publication shows that the suitable levels of glutamine (0.4–0.8%) in sea cucumbers’ diets can improve the intestinal function by improving certain digestive enzymes, the villus height and villus density in intestine [91]. Glutamine can also significantly improve nonspecific immune responses in juvenile turbot challenged by *Edwardsiella tarda* [92].

## 5. BCAAs as Biomarkers in Diseases

### 5.1. Insulin Resistance (IR)

Insulin resistance (IR) can be defined as a decline of insulin efficiency on glucose uptake and utilization. Metabolic perturbation of glucose and fatty acid oxidation caused by over-nourishment or obesity would lead to mitochondrial dysfunction resulting in IR. The restriction of fatty acid oxidation in adipose tissue of obesity is associated with elevated BCAAs catabolism in skeletal muscle and accumulation of lipid intermediates like acyl carnitines, which damaged insulin action [28,93]. Therefore, levels of acyl carnitines in serum or plasma could be taken as a marker of IR. In addition, dietary BCAAs supplementation has shown potential benefits for the metabolic profile. However, higher intakes of BCAAs may have adverse effects on development of IR [94], and higher blood BCAAs levels have been associated with IR [95]. Asghari et al. [94] revealed that high consumption of dietary BCAAs may increase risk of incident IR and can accelerate the development of metabolic abnormalities such as metabolic syndrome, and diabetes, and not associated with β-cell dysfunction and hyperinsulinemia in adults. Allam-Ndoul et al. reported that plasma BCAAs concentrations might serve as a better indicator of impaired IR in pre-diabetic state than plasma glucose levels [95]. McCormack et al. also reported that elevations in BCAAs concentrations are significantly related to obesity in children and adolescents, and may be independently predict future IR [96]. Human and rodent studies showed that these correlative relationships between BCAAs and IR may be dependent on energy homeostasis, and circulating BCAAs can be used as a potential molecular indicators of glucose uptake and insulin sensitivity [97].

A consequence of increased BCAAs levels is the activation of the mTOR/p70S6K pathway and phosphorylation of IRS-1 on multiple serine sites, contributing to IR. As a catabolic intermediate of the Val, 3-hydroxyisobutyrate (3-HIB) is secreted from muscle cells, and regulates trans-endothelial fatty acid transport and promotes lipid accumulation in muscle and leading to IR in mice. Compared with normal individuals, the level of metabolite 3-HIB is increased in muscles from mice and human with diabetes [19]. These results indicated that 3-HIB can serve as a signaling metabolite in disease like diabetes and IR. 

An overwhelming number of publications have consistently demonstrated that concentrations of BCAAs in plasma and urine are associated with IR. Intermediates derived from BCAAs breakdown rather than BCAAs themselves were recently proposed to contribute to the development of IR, and studies now explore the biomarker qualities of these metabolites. Studies of BCAA supplementation in both animals and humans [3] indicate that circulating AA may directly promote IR via disruption of insulin signaling in skeletal muscle. Furthermore, a significant association is detected between concentrations of BCAAs and adipokines including adiponectin, leptin and updated homeostasis model assessment of insulin resistance (HOMA2-IR) in the plasma of Japanese adults without diabetes [98].

### 5.2. Type 2 Diabetic Mellitus (T2DM)

Diabetic mellitus portends a poor prognosis concerning pressure overloaded heart disease. BCAAs catabolism altered in T2DM has been reported decades ago. The intake of BCAAs has potential benefits for the metabolic profile, while high intake of BCAAs may be associated with a decrease in the risk of diabetes [99]. An association investigation between cumulative consumption of BCAAs and risk of T2DM suggest higher dietary intakes of BCAAs are associated with an increased risk of T2DM in three prospective cohorts [100]. Similar associations were observed in an Asian population [101]. However, the relationship between BCAAs and T2DM inducing deterioration of pressure overloaded heart disease remains controversial. The particular effect of BCKA on myocardial injury induced by pressure overloaded showed that BCKA could decrease cell survival and increase apoptosis dependently via inactivation of mTORC2-Akt pathway [62]. Serum BCAAs profile has implications for somatic mTORC1 activity in humans and mice, while not required for improved metabolism in diabetes [102]. Dietary protein sources are closely related to body adiposity and metabolic health, e.g., obesity, T2DM and IR. Changing dietary protein sources to a vegan diet supplemented with fish could decline the number of plasma BCAAs that have been linked to the risk of diabetes and obesity, and the high level of BCAAs in humans may be a marker for dietary patterns associated with diabetes and obesity [103]. 

Many investigations indicate that BCAAs have the potential to predict diabetes development. Plasma-free amino acid (PFAA) profiles, particularly the levels of BCAAs, are altered before the development of T2DM, and significantly associated with a future diagnosis of diabetes mellitus [104]. These PFAA alterations might predominantly result from the metabolic shift caused by early pathogenesis of diabetes. Research investigated the metabolite profiles including AAs, amines, and other polar metabolites among 2422 normoglycemic individuals in which 201 developed diabetes during the following 12 years. The results show that three BCAAs and two aromatic AAs (tyrosine and phenylalanine) had highly significant associations with future diabetes. These findings underscore the potential importance of AAs profiles aiding in diabetes risk assessment [105]. A recent publication indicated that increased plasma level of 3-HIB is a marker of future risk of T2DM, and 3-HIB may be important for the regulation of metabolic flexibility in heart and muscles [106].

### 5.3. Cancer

PI3K/Akt/mTOR pathway can be used as a target for cancer therapy [106,107]. The process of proliferation and growth of cancer cells need to acquire essential nutrients from the tumor microenvironment [108]. Even though the conditions of nutrient and oxygen availability are poor, cancer cells can also use them to maintain biomass and survival [109]. As essential nutrients for cancer growth, BCAAs are utilized by tumors in various biosynthetic pathways and as an energy source of cancer cells [110]. BCAAs metabolism and expression of BCAAs associated with metabolic enzymes are closely related to oncogenic mutations and cancer tissue-of-origin. The cytosolic branched-chain aminotransferase 1 (BCAT1), a BCAAs metabolic enzyme, has emerged as an important prognostic cancer marker, and metabolism of BCAAs also has a potential as target therapies for development of new cancer [110]. The BCAAs levels of plasma and tissue are increased in breast cancer, which is accompanied by the elevated expression of BCAT1. Overexpression of BCAT1 shows that BCAAs catabolism is activated in human breast cancer, and opposing results are observed that the knockdown of BCAT1 can inhibit breast cancer cell growth by activating the mTOR, but not AMPK or SIRT1, signaling mediating mitochondrial biogenesis and function [111].

### 5.4. Cardiovascular Diseases (CVDs)

Cardiovascular disease (CVDs), also called cardiovascular and cerebrovascular disease, risk is increased in overweight or obese individuals. Nevertheless, not all overweight/obese subjects will develop the CVDs, and some overweight/obese people remain cardiometabolically healthy, and normal-weight persons develop CVDs. Defect of BCAAs catabolism is associated with cardiovascular diseases [112,113]. A recent publication investigated the potential of BCAAs to identify an increased CVDs risk in 666 adults and juveniles classified as lean, overweight or obese, which indicated elevated serum levels of BCAAs, especially Val and Leu, are proposed as a cardiometabolic risk marker irrespective of body mass index category [114]. A study of Zhen et al. [26] suggests that mTORC1 is involved in the regulation of cardiac BCAAs catabolism. It is concluded in this report that the Leu administration could significantly decrease the cardiac concentrations of Ile and Val, and an activation of the mTOR system and/or abnormal yet enigmatic BCAAs metabolic pathways may be involved. As is well known, BCAAs-rich diets have beneficial health effects on metabolic health. However, several studies have reported that increased BCAAs levels are significant correlated with CVDs risk [115,116]. Nakamura et al. [4] reported that the AAs levels in diabetic patients were strongly associated with hyperinsulinemia and hypoadiponectinemia, which might become risk evaluation factors for the development of CVDs. Gilstrap et al. have showed that serum BCAAs are independently associated with increased carotid intima-media thickness (cIMT), and this association would open a new understanding of atherosclerosis and the risk assessment of CVDs [115]. 

## 6. Conclusions

BCAAs play critical roles of nutrition physiological functions in glucose and lipid metabolism, protein synthesis, as well as intestinal health and immune. Catabolism and balance of BCAAs are closely associated with health and disease, and PI3K-AKT-mTOR as a main nutrient-sensitive signaling pathway mediates the BCAAs metabolism and disease progression. Moreover, BCAAs and their derivatives can serve as the potential biomarkers of diseases such as IR, T2DM, cancer, and CVDs. Further research is warranted to investigate whether the plasma BCAAs and its metabolite measurements can help predict diseases including atherosclerosis or CVDs and to elucidate the biological mechanisms of the identified associations between the levels of plasma AAs and various CVDs risk factors. PI3K-AKT-mTOR pathway could be used as a potential target for diseases by BCAAs regulation.

## References

[1] Jewell J.L., Russell R.C., Guan K.L. (2013). Amino acid signalling upstream of mTOR. Nat. Rev. Mol. Cell Biol..

[2] Lynch C.J., Adams S.H. (2014). Branched-chain amino acids in metabolic signalling and insulin resistance. Nat. Rev. Endocrinol..

[3] Newgard C.B., An J., Bain J.R., Muehlbauer M.J., Stevens R.D., Lien L.F., Haqq A.M., Shah S.H., Arlotto M., Slentz C.A. (2009). A branched-chain amino acid-related metabolic signature that differentiates obese and lean humans and contributes to insulin resistance. Cell Metab..

[4] Nakamura H., Jinzu H., Nagao K., Noguchi Y., Shimba N., Miyano H., Watanabe T., Iseki K. (2014). Plasma amino acid profiles are associated with insulin, C-peptide and adiponectin levels in type 2 diabetic patients. Nutr. Diabetes.

[5] Ruiz-canela M., Toledo E., Clish C.B., Hruby A., Liang L., Salas-Salvadó J., Razquin C., Corella D., Estruch R., Ros E. (2016). Plasma branched-chain amino acids and incident cardiovascular disease in the predimed trial. Clin. Chem..

[6] Batcha B.C., Hylanda K., Svetkey L.P. (2014). Branch chain amino acids: Biomarkers of health and disease. Curr. Opin. Clin. Nutr. Metab. Care.

[7] O’Donnella J.S., Massi D., Teng M.W.L., Mandala M. (2018). PI3K-AKT-mTOR inhibition in cancer immunotherapy, redux. Semin. Cancer Biol..

[8] Dey N., De P., Leyland-Jones B. (2017). PI3K-AKT-mTOR inhibitors in breast cancers: From tumor cell signaling to clinical trials. Pharmacol. Ther..

[9] Tato I., Bartrons R., Ventura F., Rosa J.L. (2011). Amino acids activate mammalian target of rapamycin complex 2 (mTORC2) via PI3K/Akt signaling. J. Biol. Chem..

[10] Adeva-Andany M.M., López-Maside L., Donapetry-García C., Fernández-Fernández C., Sixto-Lea C. (2017). Enzymes involved in branched-chain amino acid metabolism in humans. Amino Acids.

[11] Sperringer J.E., Addington A., Hutson S.M. (2017). Branched-chain amino acids and brain metabolism. Neurochem. Res..

[12] Fan P., Li L., Rezaei A., Eslamfam S., Che D., Ma X. (2015). Metabolites of dietary protein and peptides by intestinal microbes and their impacts on gut. Curr. Protein Pept. Sci..

[13] Boutry C., El-Kadi S.W., Suryawan A., Wheatley S.M., Orellana R.A., Kimball S.R., Nguyen H.V., Davis T.A. (2013). Leucine pulses enhance skeletal muscle protein synthesis during continuous feeding in neonatal pigs. Am. J. Physiol. Endocrinol. Metab..

[14] Zheng L., Zuo F., Zhao S., He P., Wei H., Xiang Q., Pang J., Peng J. (2017). Dietary supplementation of branched-chain amino acids increases muscle net amino acid fluxes through elevating their substrate availability and intramuscular catabolism in young pigs. Br. J. Nutr..

[15] Bröer S., Bröer A. (2017). Amino acid homeostasis and signalling in mammalian cells and organisms. Biochem. J..

[16] Zhenyukh O., Civantos E., Ruiz-Ortega M., Sánchez M.S., Vázquez C., Peiró C., Egido J., Mas S. (2017). High concentration of branched-chain amino acids promotes oxidative stress, inflammation and migration of human peripheral blood mononuclear cells via mtorc1 activation. Free Radic. Biol. Med..

[17] Liu K.A., Lashinger L.M., Rasmussen A.J., Hursting S.D. (2014). Leucine supplementation differentially enhances pancreatic cancer growth in lean and overweight mice. Cancer Metab..

[18] Neishabouri S.H., Hutson S.M., Davoodi J. (2015). Chronic activation of mTOR complex 1 by branched chain amino acids and organ hypertrophy. Amino Acids.

[19] Jang C., Oh S.F., Wada S., Rowe G.C., Liu L., Chan M.C., Rhee J., Hoshino A., Kim B., Ibrahim A. (2016). A branched chain amino acid metabolite drives vascular transport of fat and causes insulin resistance. Nat. Med..

[20] Laplante M., Sabatini D.M. (2012). mTOR signaling in growth control and disease. Cell.

[21] Bar-Peled L., Sabatini D.M. (2014). Regulation of mTORC1 by amino acids. Trends Cell Biol..

[22] Sonnet D.S., O’Leary M.N., Gutierrez M.A., Nguyen S.M., Mateen S., Hsu Y., Mitchell K.P., Lopez A.J., Vockley J., Kennedy B.K. (2016). Metformin inhibits branched chain amino acid (BCAAs) derived ketoacidosis and promotes metabolic homeostasis in msud. Sci. Rep..

[23] Soomro R.N., Hu R., Qiao Y., El-Hack M.E.A., Abbasi I.H.R., Mohamed M.A.E., Alagawany M., Yang X., Yao J., Dhama K. (2017). Effects of dietary protein sources and amino acid balance on growth performance, intestinal permeability and morphology in broiler chickens. Int. J. Pharm..

[24] Nofal M., Zhang K., Han S., Rabinowitz J.D. (2017). mTOR inhibition restores amino acid balance in cells dependent on catabolism of extracellular protein. Mol. Cell.

[25] Wiltafsky M.K., Pfaffl M.W., Roth F.X. (2010). The effects of branched-chain amino acid interactions on growth performance, blood metabolites, enzyme kinetics and transcriptomics in weaned pigs. Br. J. Nutr..

[26] Zhen H., Kitaura Y., Kadota Y., Ishikawa T., Kondo Y., Xu M., Morishita Y., Ota M., Ito T., Shimomura Y. (2016). mTORC1 is involved in the regulation of branched-chain amino acid catabolism in mouse heart. FEBS Open Bio.

[27] Stipanuk M.H. (2007). Leucine and protein synthesis: mTOR and beyond. Nutr. Rev..

[28] Sheriff D.S., Younis M.Y.G., Elshaari F.A., Mohamed N.A., Kuwaila H.I.A.E., Abdalla S.A.S., Elfaghi R. (2014). A perspective on interaction between lipid and branched chain amino acids (BCAAs) in developing insulin resistance. Med. J..

[29] Herman M.A., She P., Peroni O.D., Lynch C.J., Kahn B.B. (2010). Adipose tissue branched chain amino acid (BCAAs) metabolism modulates circulating BCAAs levels. J. Biol. Chem..

[30] Cummings N.E., Williams E.M., Kasza I., Konon E.N., Schaid M.D., Schmidt B.A., Poudel C., Sherman D.S., Yu D., Arriola Apelo S.I. (2017). Restoration of metabolic health by decreased consumption of branched-chain amino acids. J. Physiol..

[31] Long B., Muhamad R., Yan G., Yu J., Fan Q., Wang Z., Li X., Purnomoadi A., Achmadi J., Yan X. (2016). Quantitative proteomics analysis reveals glutamine deprivation activates fatty acid β-oxidation pathway in HepG2 cells. Amino Acids.

[32] Liu Y., Dong W., Shao J., Wang Y., Zhou M., Sun H. (2017). Branched-chain amino acid negatively regulates KLF15 expression via PI3K-AKT pathway. Front. Physiol..

[33] Fruman D.A., Chiu H., Hopkins B.D., Bagrodia S., Cantley L.C., Abraham R.T. (2017). The PI3K pathway in human disease. Cell.

[34] Ma X., Zhang S., He L., Rong Y., Brier L.W., Sun Q., Liu R., Fan W., Chen S., Yue Z. (2017). MTORC1-mediated NRBF2 phosphorylation functions as a switch for the class III PtdIns3K and autophagy. Autophagy.

[35] Doi M., Yamaoka I., Fukunaga T., Nakayama M. (2003). Isoleucine, a potent plasma glucose-lowering amino acid, stimulates glucose uptake in C2C12, myotubes. Biochem. Biophys. Res. Commun..

[36] Doi M., Yamaoka I., Nakayama M., Mochizuki S., Sugahara K., Yoshizawa F. (2005). Isoleucine, a blood glucose-lowering amino acid, increases glucose uptake in rat skeletal muscle in the absence of increases in AMP-activated protein kinase activity. J. Nutr..

[37] Nishimura J., Masaki T., Arakawa M., Seike M., Yoshimatsu H. (2010). Isoleucine prevents the accumulation of tissue triglycerides and upregulates the expression of PPARα and uncoupling protein in diet-induced obese mice. J. Nutr..

[38] Du Y., Meng Q., Zhang Q., Guo F. (2012). Isoleucine or valine deprivation stimulates fat loss via increasing energy expenditure and regulating lipid metabolism in WAT. Amino Acids.

[39] Grahame Hardie D. (2014). AMP-activated protein kinase: A key regulator of energy balance with many roles in human disease. J. Intern. Med..

[40] Bai J., Greene E., Li W., Kidd M.T., Dridi S. (2015). Branched-chain amino acids modulate the expression of hepatic fatty acid metabolism-related genes in female broiler chickens. Mol. Nutr. Food Res..

[41] Duan Y., Li F., Wang W., Guo Q., Wen C., Yin Y. (2017). Alteration of muscle fiber characteristics and the AMPK-SIRT1-PGC-1α axis in skeletal muscle of growing pigs fed low-protein diets with varying branched-chain amino acid ratios. Oncotarget.

[42] Ehling S., Reddy T.M. (2015). Direct analysis of leucine and its metabolites β-hydroxy-β-methylbutyric acid, α-ketoisocaproic acid, and α-hydroxyisocaproic acid in human breast milk by liquid chromatography-mass spectrometry. J. Agric. Food Chem..

[43] Dong W., Zhou M., Mei D., Pan B., Liu Y., Jing S., Gu X., Huang Y., Li G., Wang Y. (2016). Keto acid metabolites of branched-chain amino acids inhibit oxidative stress-induced necrosis and attenuate myocardial ischemia-reperfusion injury. J. Mol. Cell Cardiol..

[44] Hasselgren P.O. (2014). Beta-Hydroxy-beta-methylbutyrate (HMB) and prevention of muscle wasting. Metabolism.

[45] Medras Z.J.H., El-Sayed N.M., Zaitone S.A., Toraih E.A., Samie M.M., Moustafa Y.M. (2017). Glutamine up-regulates pancreatic sodium-dependent neutral aminoacid transporter-2 and mitigates islets apoptosis in diabetic rats. Pharmacol. Rep..

[46] Ma X., Han M., Li D., Hu S., Gilbreath K.R., Bazer F.W., Wu G. (2017). L-Arginine promotes protein synthesis and cell growth in brown adipocyte precursor cells via the mTOR signal pathway. Amino Acids.

[47] Fan L., Hsieh P.N., Sweet D.R., Jain M.K. (2017). Krüppel-like factor 15: Regulator of BCAAs metabolism and circadian protein rhythmicity. Pharmacol. Res..

[48] Kimball S.R., Jefferson L.S. (2006). New functions for amino acids: Effects on gene transcription and translation. Am. J. Clin. Nutr..

[49] Columbus D.A., Fiorotto M.L., Davis T.A. (2015). Leucine is a major regulator of muscle protein synthesis in neonates. Amino Acids.

[50] López N., Sánchez J., Palou A., Serra F. (2018). Gender-associated impact of early leucine supplementation on adult predisposition to obesity in rats. Nutrients.

[51] Anthony J.C., Lang C.H., Crozier S.J., Anthony T.G., MacLean D.A., Kimball S.R., Jefferson L.S. (2002). Contribution of insulin to the translational control of protein synthesis in skeletal muscle by leucine. Am. J. Physiol..

[52] Crozier S.J., Kimball S.R., Emmert S.W., Anthony J.C., Jefferson L.S. (2005). Oral leucine administration stimulates protein synthesis in rat skeletal muscle. J. Nutr..

[53] Churchward-Venne T.A., Breen L., Di Donato D.M., Hector A.J., Mitchell C.J., Moore D.R., Stellingwerff T., Breuille D., Offord E.A., Baker S.K. (2014). Leucine supplementation of a low-protein mixed macronutrient beverage enhances myofibrillar protein synthesis in young men: A double-blind, randomized trial. Am. J. Clin. Nutr..

[54] Wyant G.A., Aburemaileh M., Wolfson R.L., Chen W.W., Freinkman E., Danai L.V., Heiden M.G.V., Sabatini D.M. (2017). mTORC1 activator SLC38A9 is required to efflux essential amino acids from lysosomes and use protein as a nutrient. Cell.

[55] Anthony J.C., Anthony T.G., Kimball S.R., Vary T.C., Jefferson L.S. (2010). Orally administered leucine stimulates protein synthesis in skeletal muscle of postabsorptive rats in association with increased eIF4F formation. J. Nutr..

[56] Davis T.A., Fiorotto M.L. (2009). Regulation of muscle growth in neonates. Curr. Opin. Nutr. Metab. Care.

[57] He L., Eslamfam S., Ma X., Li D. (2016). Autophagy and the nutritional signaling pathway. Front. Agric. Sci. Eng..

[58] Jackman S.R., Witard O.C., Philp A., Wallis G.A., Baar K., Tipton K.D. (2017). Branched-chain amino acid ingestion stimulates muscle myofibrillar protein synthesis following resistance exercise in humans. Front. Physiol..

[59] Zhang S., Ren M., Zeng X., He P., Ma X., Qiao S. (2014). Leucine stimulates ASCT2 amino acid transporter expression in porcine jejunal epithelial cell line (IPEC-J2) through PI3K/AKT/mTOR and ERK signaling pathways. Amino Acids.

[60] Wolfson R.L., Chantranupong L., Saxton R.A., Shen K., Scaria S.M., Cantor J.R., Sabatini D.M. (2016). Sestrin2 is a leucine sensor for the mTORC1 pathway. Science.

[61] Han J.M., Jeong S.J., Park M.C., Kim G., Kwon N.H., Kim H.K., Ha S.H., Ryu S.H., Kim S. (2012). Leucyl-tRNA synthetase is an intracellular leucine sensor for the mTORC1-signaling pathway. Cell.

[62] Guo X., Huang C., Lian K., Wang S., Zhao H., Yan F., Zhang X., Zhang J., Xie H., An R., Tao L. (2016). BCKA down-regulates mTORC2-Akt signal and enhances apoptosis susceptibility in cardiomyocytes. Biochem. Biophys. Res. Commun..

[63] Wilkinson D.J., Hossain T., Limb M.C., Phillips B.E., Lund J., Williams J.P., Brook M.S., Cegielski J., Philp A., Ashcroft S. (2017). Impact of the calcium form of β-hydroxy-β-methylbutyrate upon human skeletal muscle protein metabolism. Clin. Nutr..

[64] Girón M.D., Vílchez J.D., Salto R., Manzano M., Sevillano N., Campos N., Argilés J.M., Rueda R., López-Pedrosa J.M. (2016). Conversion of leucine to β-hydroxy-β-methylbutyrate by α-keto isocaproate dioxygenase is required for a potent stimulation of protein synthesis in l6 rat myotubes. J. Cachexia Sarcopenia Muscle.

[65] Wheatley S.M., El-Kadi S.W., Suryawa A., Boutry C., Orellana R.A., Nguyen H.V., Davis S.R., Davis T.A. (2014). Protein synthesis in skeletal muscle of neonatal pigs is enhanced by administration of beta-hydroxy-beta-methylbutyrate. Am. J. Physiol. Endocrinol. Metab..

[66] Kao M., Columbus D.A., Suryawan A., Steinhoff-Wagner J., Hernandez-Garcia A., Nguyen H.V., Fiorotto M.L., Davis T.A. (2016). Enteral β-hydroxy-β-methylbutyrate supplementation increases protein synthesis in skeletal muscle of neonatal pigs. Am. J. Physiol. Endocrinol. Metab..

[67] Gerlinger-Romero F., Guimarães-Ferreira L., Yonamine C.Y., Salgueiro R.B., Nunes M.T. (2017). Effects of beta-hydroxy-beta-methylbutyrate (HMB) on the expression of ubiquitin ligases, protein synthesis pathways and contractile function in extensor digitorum longus (DEL) of fed and fasting rats. J. Physiol. Sci..

[68] Aversa Z., Bonetto A., Costelli P., Minero V.G., Penna F., Baccino F.M., Lucia S., Rossi Faelli F., Muscaritoli M. (2011). β-hydroxy-β-methylbutyrate (HMB) attenuates muscle and body weight loss in experimental cancer cachexia. Int. J. Oncol..

[69] Van Zanten A.R. (2015). Glutamine and antioxidants: Status of their use in critical illness. Curr. Opin. Clin. Nutr. Metab. Care.

[70] Flint H.J., Scott K.P., Louis P., Duncan S.H. (2012). The role of the gut microbiota in nutrition and health. Nat. Rev. Gastroenterol. Hepatol..

[71] Kau A.L., Ahern P.P., Griffin N.W., Goodman A.L., Gordon J.I. (2011). Human nutrition, the gut microbiome and immune system. Nature.

[72] Garrett W.S., Gordon J.I., Glimcher L.H. (2010). Homeostasis and inflammation in the intestine. Cell.

[73] He L., Han M., Farrar S., Ma X. (2017). Impacts and regulation of dietary nutrients on gut microbiome and immunity. Protein Pept. Lett..

[74] Mcgaha T.L., Huang L., Lemos H., Metz R., Mautino M., Prendergast G.C., Mellor A.L. (2012). Amino acid catabolism: A pivotal regulator of innate and adaptive immunity. Immunol. Rev..

[75] Ren M., Zhang S.H., Zeng X.F., Liu H., Qiao S.Y. (2015). Branched-chain amino acids are beneficial to maintain growth performance and intestinal immune-related function in weaned piglets fed protein restricted diet. Asian-australas. J. Anim. Sci..

[76] Ren M., Zhang S., Liu X., Li S., Mao X., Zeng X., Qiao S. (2016). Different lipopolysaccharide branched-chain amino acids modulate porcine intestinal endogenous β-defensin expression through the Sirt1/ERK/90RSK pathway. J. Agric. Food Chem..

[77] Liu H., Wang J., He T., Becker S., Zhang G., Li D., Ma X. (2018). Butyrate: A double-edged sword for health?. Adv. Nutr..

[78] Jiang W.D., Deng Y.P., Liu Y., Qu B., Jiang J., Kuang S.Y., Tang L., Tang W., Wu P., Zhang Y., Zhou X., Feng L. (2015). Dietary leucine regulates the intestinal immune status, immune-related signalling molecules and tight junction transcript abundance in grass carp (*Ctenopharyngodon idella*). Aquaculture.

[79] Chen J., Li Y., Tian Y., Huang C., Li D., Zhong Q., Ma X. (2015). Interaction between microbes and host intestinal health: Modulation by dietary nutrients and gut-brain-endocrine-immune axis. Curr. Protein Pept. Sci..

[80] Ma N., Tian Y., Wu Y., Ma X. (2017). Contributions of the interaction between dietary protein and gut microbiota to intestinal health. Curr. Protein Pept. Sci..

[81] Zhao J., Feng L., Liu Y., Jiang W., Wu P., Jiang J., Zhang Y., Zhou X. (2014). Effect of dietary isoleucine on the immunity, antioxidant status, tight junctions and microflora in the intestine of juvenile jian carp (*Cyprinus carpio*, var. jian). Fish Shellfish Immunol..

[82] Calder P.C. (2006). Branched-chain amino acid and immunity. J. Nutr..

[83] De Simone R., Vissicchio F., Mingarelli C., De Nuccio C., Visentin S., Ajmone-Cat M.A., Minghetti L. (2013). Branched-chain amino acids influence the immune properties of microglial cells and their responsiveness to pro-inflammatory signals. Biochim. Biophys. Acta.

[84] Ma N., Guo P., Zhang J., He T., Kim S.W., Zhang G., Ma X. (2018). Nutrients mediate intestinal bacteria-mucosal immune crosstalk. Front. Immunol..

[85] Nakamura I. (2014). Impairment of innate immune responses in cirrhotic patients and treatment by branched-chain amino acids. World J. Gastroenterol..

[86] Rivas-Santiago C., Rivas-Santiago B., León D., Castañeda-Delgado J., Hernández P.R. (2011). Induction of β-efensins by L-isoleucine as novel immunotherapy in experimental murine tuberculosis. Clin. Exp. Immunol..

[87] Bassit R.A., Sawada L.A., Bacurau R.F., Navarro F., Martins E., Santos R.V., Caperuto E.C., Rogeri P., Rosa L.F.C. (2002). Branched-chain amino acid supplementation and the immune response of long-distance athletes. Nutrition.

[88] Kakazu E., Kanno N., Ueno Y., Shimosegawa T. (2007). Extracellular branched-chain amino acids, especially valine, regulate maturation and function of monocyte-derived dendritic cells. J. Immunol..

[89] Zhao J., Liu Y., Jiang J., Wu P., Jiang W., Li S., Tang L., Kuang S., Feng L., Zhou X. (2013). Effects of dietary isoleucine on the immune response, antioxidant status and gene expression in the head kidney of juvenile jian carp (*Cyprinus carpio* var. jian). Fish Shellfish Immunol..

[90] Fan J., Wu L., Li G., Tao S., Sheng Z., Meng Q., Li F., Yu L., Li L. (2015). Effects of enteral nutrition with parenteral glutamine supplementation on the immunological function in septic rats. Br. J. Nutr..

[91] Yu H., Gao Q., Dong S., Lan Y., Ye Z., Wen B. (2016). Regulation of dietary glutamine on the growth, intestinal function, immunity and antioxidant capacity of sea cucumber *Apostichopus japonicus* (selenka). Fish Shellfish Immunol..

[92] Zhang K., Mai K., Xu W., Liufu Z., Zhang Y., Peng M., Chen J., Ai Q. (2017). Effects of dietary arginine and glutamine on growth performance, nonspecific immunity, and disease resistance in relation to arginine catabolism in juvenile turbot (*Scophthalmus maximus* L.). Aquaculture.

[93] Guillet C., Delcourt I., Rance M., Giraudet C., Walrand S., Bedu M., Duche P., Boirie Y.J. (2009). Changes in basal and insulin and amino acid response of whole body and skeletal muscle proteins in obese men. J. Clin. Endocrinol. Metab..

[94] Asghari G., Farhadnejad H., Teymoori F., Mirmiran P., Tohidi M., Azizi F. (2017). High dietary intakes of branched-hain amino acids is associated with increased risk of insulin resistance in adults. J. Diabetes.

[95] Allam-Ndoul B., Guénard F., Garneau V., Barbier O., Pérusse L., Vohl M. (2015). Associations between branched chain amino acid levels, obesity and cardiometabolic complications. Integr. Obes. Diabetes.

[96] McCormack S.E., Shaham O., McCarthy M.A., Deik A.A., Wang T.J., Gerszten R.E., Clish C.B., Mootha V.K., Grinspoon S.K., Fleischman A. (2013). Circulating branched-chain amino acid concentrations are associated with obesity and future insulin resistance in children and adolescents. Pediatr. Obes..

[97] Gannon N.P., Schnuck J.K., Vaughan R.A. (2018). BCAA metabolism and insulin sensitivity-dysregulated by metabolic status?. Mol. Nutr. Food Res..

[98] Katagiri R., Goto A., Budhathoki S., Yamaji T., Yamamoto H., Kato Y., Iwasaki M., Tsugane S. (2018). Association between plasma concentrations of branched-chain amino acids and adipokines in Japanese adults without diabetes. Sci. Rep..

[99] Nagata C., Nakamura K., Wada K., Tsuji M., Tamai Y., Kawachi T. (2013). Branched-chain amino acid intake and the risk of diabetes in a Japanese community: the Takayama study. Am. J. Epidemiol..

[100] Zheng Y., Li Y., Qi Q., Hruby A., Manson J.E., Willett W.C., Wolpin B.M., Hu F.B., Qi L. (2016). Cumulative consumption of branched-chain amino acids and incidence of type 2 diabetes. Int. J. Epidemiol..

[101] Xu F., Tavintharan S., Sum C.F., Woon K., Lim S.C., Ong C.N. (2013). Metabolic signature shift in type 2 diabetes mellitus revealed by mass spectrometry-based metabolomics. J. Clin. Endocrinol. Metab..

[102] Maida A., Chan J., Sjøberg K.A., Zota A., Schmoll D., Kiens B., Herzig S., Rose A.J. (2017). Repletion of branched chain amino acids reverses mtorc1 signaling but not improved metabolism during dietary protein dilution. Mol. Metab..

[103] Elshorbagy A., Jernerén F., Basta F., Basta C., Turner C., Khaled M., Refsum H. (2017). Amino acid changes during transition to a vegan diet supplemented with fish in healthy humans. Eur. J. Nutr..

[104] Wang T.J., Larson M.G., Vasan R.S., Cheng S., Rhee E.P., McCabe E., Lewis G.D., Fox C.S., Jacques P.F., Fernandez C. (2011). Metabolite profiles and the risk of developing diabetes. Nat. Med..

[105] Mardinoglu A., Gogg S., Lotta L.A., Stančákováe A., Nerstedt A., Boren J., Blüher M., Ferrannini E., Langenberg C., Wareham N.J. (2018). Elevated plasma levels of 3-Hydroxyisobutyric acid are associated with incident Type 2 diabetes. EBioMedicine.

[106] Morgensztern D., Mcleod H.L. (2005). PI3K/AKT/mTOR pathway as a target for cancer therapy. Anticancer Drugs.

[107] Morgan T.M., Koreckij T.D., Corey E. (2009). Targeted therapy for advanced prostate cancer: inhibition of the PI3K/AKT/mTOR pathway. Curr. Cancer Drug Targets.

[108] Dibble C.C., Cantley L.C. (2015). Regulation of mtorc1 by pi3k signaling. Trends Cell Biol..

[109] Reina-Campos M., Moscat J., Diaz-Meco M. (2017). Metabolism shapes the tumor microenvironment. Curr. Opin. Cell Biol..

[110] Deberardinis R.J., Chandel N.S. (2016). Fundamentals of cancer metabolism. Sci. Adv..

[111] Ananieva E.A., Wilkinson A.C. (2018). Branched-chain amino acid metabolism in cancer. Curr. Opin. Clin. Nutr. Metab. Care.

[112] Zhang L., Han J. (2017). Branched-chain amino acid transaminase 1 (BCAT1) promotes the growth of breast cancer cells through improving mTOR-mediated mitochondrial biogenesis and function. Biochem. Biophys. Res. Commun..

[113] Shah S.H., Bain J.R., Muehlbauer M.J., Stevens R.D., Crosslin D.R., Haynes C., Dungan J., Newby L.K., Hauser E.R., Ginsburg G.S. (2010). Association of a peripheral blood metabolic profile with coronary artery disease and risk of subsequent cardiovascular events. Circ. Cardiovasc. Genet..

[114] Sun H., Olson K.C., Gao C., Prosdocimo D.A., Zhou M., Wang Z., Jeyaraj D., Youn J., Ren S., Liu Y. (2016). Catabolic defect of branched-chain amino acids promotes heart failure. Circulation.

[115] Mangge H., Zelzer S., Prüller F., Schnedl W.J., Weghuber D., Enko D., Bergsten P., Haybaeck J., Meinitzer A. (2016). Branched-chain amino acids are associated with cardiometabolic risk profiles found already in lean, overweight and obese young. J. Nutr. Biochem..

[116] Gilstrap L.G., Wang T.J. (2012). Biomarkers and cardiovascular risk assessment for primary prevention: An update. Clin. Chem..

